# Thyroid hormone and anti-apoptosis in tumor cells

**DOI:** 10.18632/oncotarget.4023

**Published:** 2015-05-23

**Authors:** Hung-Yun Lin, Gennadi V. Glinsky, Shaker A. Mousa, Paul J. Davis

**Affiliations:** ^1^ PhD Program for Cancer Biology and Drug Discovery, College of Medical Science and Technology, Taipei, Taiwan; ^2^ Taipei Cancer Center, Taipei Medical University, Taipei, Taiwan; ^3^ Stanford University, Palo Alto, CA, USA; ^4^ Pharmaceutical Research Institute, Albany College of Pharmacy and Health Sciences, Albany, NY, USA; ^5^ Department of Medicine, Albany Medical College, Albany, NY, USA

**Keywords:** thyroid hormone, integrin αvβ3, resveratrol, tetrac, apoptosis

## Abstract

The principal secretory product of the thyroid gland, L-thyroxine (T_4_), is anti-apoptotic at physiological concentrations in a number of cancer cell lines. Among the mechanisms of anti-apoptosis activated by the hormone are interference with the Ser-15 phosphorylation (activation) of p53 and with TNFα/Fas-induced apoptosis. The hormone also decreases cellular abundance and activation of proteolytic caspases and of *BAX* and causes increased expression of X-linked inhibitor of apoptosis (*XIAP*). The anti-apoptotic effects of thyroid hormone largely are initiated at a cell surface thyroid hormone receptor on the extracellular domain of integrin αvβ3 that is amply expressed and activated in cancer cells. Tetraiodothyroacetic acid (tetrac) is a T_4_ derivative that, in a model of resveratrol-induced p53-dependent apoptosis in glioma cells, blocks the anti-apoptotic action of thyroid hormone, permitting specific serine phosphorylation of p53 and apoptosis to proceed. In a nanoparticulate formulation limiting its action to αvβ3, tetrac modulates integrin-dependent effects on gene expression in human cancer cell lines that include increased expression of a panel of pro-apoptotic genes and decreased transcription of defensive anti-apoptotic *XIAP* and *MCL1* genes. By a variety of mechanisms, thyroid hormone (T_4_) is an endogenous anti-apoptotic factor that may oppose chemotherapy-induced apoptosis in αvβ3-expressing cancer cells. It is possible to decrease this anti-apoptotic activity pharmacologically by reducing circulating levels of T_4_ or by blocking effects of T_4_ that are initiated at αvβ3.

## INTRODUCTION

Thyroid hormone as L-thyroxine (T_4_) is the major secretory product of the normal thyroid gland. It is converted to 3,5,3′-triiodo-L-thyronine (T_3_) by deiodination to effect genomic hormonal actions that require nuclear receptors for thyroid hormone (TRs) [1]. These actions in normal cells involve transcription of a large number of genes whose transcriptional products regulate a large number of metabolic processes essential to normal cell function. Nuclear receptors may reside in nonnuclear compartments, such as cytoplasm, and undergo nuclear importation that may be thyroid hormone-directed [2, 3]. Or, they may interact as a plasma membrane receptor with thyroid hormone [4, 5], to participate in nongenomic events that may not require nuclear uptake of TR.

In dividing endothelial cells, tumor cells, osteoclasts and certain other cells, the plasma membrane bears in large quantities a structural protein, integrin αvβ3, whose extracellular domain contains a specific receptor for thyroid hormone [6, 7]. This receptor site is not structurally related to the binding site for iodothyronines on TRs. The functions of this receptor on the integrin are distinct from those of the nuclear receptors. However, the hormone-binding site on αvβ3 may support nuclear uptake (trafficking) of cytoplasmic TR, phosphorylation of TRs and the formation of intranuclear complexes of coactivator proteins that are relevant to genomic hormonal actions involving TR-regulated genes [8]. At the integrin αvβ3 receptor for thyroid hormone, T_4_ and T_3_ initiate complex pro-angiogenic activities that may include transcription of vascular growth factor genes [9–12], nontranscriptional modulation of the function of vascular growth factor receptors adjacent to αvβ3 [13] and enhanced endothelial cell motility [14]. The thyroid hormone receptor on the integrin also has functions in neocortex expansion in developing brain [15], in determining lung smooth muscle phenotype [16] and in regulating macrophage function [17].

The thyroid hormone receptor site on αvβ3 on tumor cells and endothelial cells provides important support for tumor-related angiogenesis, a function that is obviously undesirable in the clinical setting. We have proposed that these pro-angiogenic activities of iodothyronines based at αvβ3 may limit the effectiveness of anti-angiogenic therapy in cancer patients [14, 18], and we have shown in vascularization models such as the chick chorioallantoic membrane (CAM) [18] that pharmacologic or immunologic inhibition of thyroid hormone action at αvβ3 profoundly reduces angiogenic activity of the hormone. The integrin is expressed or activated only limitedly in non-malignant cells and endothelial cells that are not undergoing cell division.

At the integrin thyroid hormone receptor, T_4_ is a proliferative factor as well for a number of human cancer cell lines that have been studied *in vitro* [19–22]. The role of T_4_ exceeds that of T_3_ in this regard, in that these hormones are equipotent stimulators of tumor cell proliferation, but the circulating levels of free T_3_ are lower than those of T_4_. Further, in a clinical experience involving pharmacologic induction of the euthyroid hypothyroxinemic state in advanced cancer patients, maintenance of normal circulating T_3_ levels and euthyroidism—with substantial reduction in circulating levels of T_4_—has been associated with stabilization of the tumor or reduction in tumor size [23]. On the other hand, differentiated and undifferentiated PC12 (pheochromocytoma) cells distinguish between T_4_ and T_3_ at αvβ3 [24], consistent with the existence of subspecialized hormone-binding domains of the receptor on the integrin [25].

**Figure 1:**
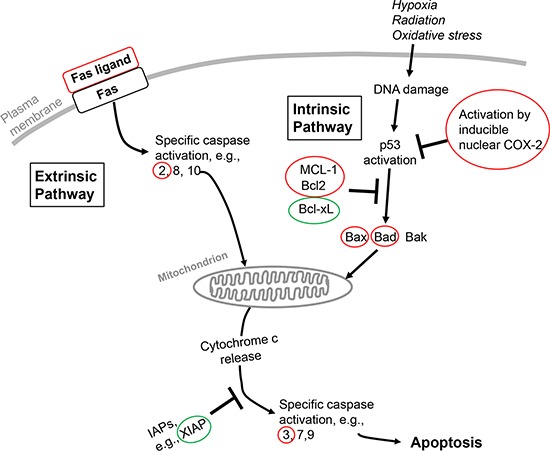
Schematic overview of extrinsic and intrinsic apoptosis pathways in the cell and points at which thyroid hormone in these pathways is anti-apoptotic The pathways converge at the mitochondrion and cause its permeabilization, with release of cytochrome c and consequent apoptosis. Genes or proteins circled in red or green identify loci of differential actions of thyroid hormone on these multiple factors in apoptosis that are discussed in the current review. Red identifies downregulation of the factor and green identifies upregulation. The Fas receptor is an activator of the *extrinsic pathway* by its interaction with Fas ligand. An activator of the *intrinsic pathway* is DNA damage resulting from factors such as radiation or chemotherapy. Bcl-2, B cell lymphoma-2; Bcl-xL, Bcl-2-related gene, long form; Bad, Bcl-2/Bcl-xL-associated death domain protein; Bak, Bcl-2 homologus antagonist killer protein; Bax, Bcl-2-associated X protein; IAPs, inhibitors of apoptosis; MCL-1, myeloid leukemia cell-1; XIAP, X-linked inhibitor of apoptosis.

Apoptosis is a regulated process of cell death in nonmalignant cells and is a conscientiously pursued therapeutic goal in oncology when the process can be limited to tumor cells. The tumor cell has a variety of anti-apoptotic transcriptional defenses and interacts with its microenvironment—malignant cell-cell adhesion, tumor cell-nonmalignant cell adhesion and tumor cell-extracellular matrix (ECM) interaction—to erect defenses against radiotherapy and chemotherapeutic measures that induce apoptosis [26]. The intrinsic apoptosis pathway involves induction by chemotherapy or radiation therapy of DNA damage and/or free radical generation that can initiate mitochondrial death signaling. Activated nuclear or mitochondrial p53 is an important mediator of such signaling. The extrinsic apoptosis pathway is activated by factors at the cell surface, for example, tumor necrosis factor-α (TNF-α) and Fas ligand [27]. The extrinsic and intrinsic pathways (Figure 1) converge at the mitochondrial membrane and control its porosity, but other events in apoptosis include loss of cytoskeletal structure, breakdown of the nuclear membrane and fragmentation of DNA. Thus, the latter may be a primary (inducing) event in response to radiation in the intrinsic pathway or a secondary event in the extrinsic pathway when DNases are activated. Thyroid hormone has anti-apoptotic actions on both the extrinsic and intrinsic pathways. The hormone also stabilizes the actin cytoskeleton via conversion of soluble actin to fibrous action (F-actin) [28] and reduces DNA breakdown by reducing caspase-activated DNase activity [29].

Against this background, it is not surprising that thyroid hormone is anti-apoptotic in tumor cells and in certain non-tumor cells. Anti-apoptosis may be desirable in nondividing neurons or endothelial cells, but is an obvious disadvantage in the setting of cancer management. Indeed, the anti-apoptotic activity of host thyroid hormone could contribute to chemotherapeutic resistance [30]. In this brief analysis, we examine the evidence that thyroid hormone is anti-apoptotic in cancer cells and the mechanisms that are involved. For example, thyroid hormone and hormone analogues affect the function of pro-apoptotic p53 [21]; that is, T_4_ blocks p53-dependent apoptosis and the T_4_ antagonist, and tetraiodothyroacetic acid (tetrac) and its formulations, facilitate apoptosis. Thyroid hormone and certain of its analogues also affect transcription of a variety of genes relevant to apoptosis [31–33]. This effect includes downregulation of expression of certain caspase genes [31]. Activated caspases are proteases essential to apoptosis by assuring structural disorganization of the nucleus and activation of DNAses, as well as contributing to degradation of cytoplasmic proteins. Tetrac is pro-apoptotic and acts at integrin αvβ3 to upregulate caspase gene transcription [31, 34]. The hypoxia-inducible factor-1α (*HIF1α*) gene in cancer cells is also upregulated by T_4_ [25]; the gene product has a number of defensive actions in tumor cells, including support of anti-apoptosis [35, 36]. Other examples of the actions of hormone analogues on apoptosis-modulating genes are discussed below.

We would also point out that thyroid hormone deprivation is associated, not unexpectedly, with increased apoptosis in breast cancer in the intact animal [37] and in hepatoma cells [38]. This may relate to increased expression of apoptosis-promoting *p53* described in tumors in hypothyroid animals [39].

### Anti-apoptotic actions of thyroid hormone on p53-related apoptosis

Resveratrol has been shown by a number of research groups to be pro-apoptotic. The cellular uptake and metabolism of resveratrol are rapid, underlying the abbreviated bioavailability of the systemically administered stilbene. A specific receptor for resveratrol exists on the extracellular domain integrin αvβ3 [40, 41] and is distinct from the receptor for thyroid hormone. The existence of the cell surface receptor for resveratrol explains why relatively brief exposure of tumor cells to the drug nonetheless results in apoptosis [42]. We have shown that apoptosis induced by resveratrol is p53-dependent, requires phosphorylation of Ser-15 of p53 by mitogen-activated protein kinase (MAPK, ERK1/2) and, interestingly, can involve nuclear accumulation of an inducible pool of cyclooxygenase-2 (COX-2) [43]. Constitutive expression of *COX-2* is a biomarker of tumor cell aggressiveness [44], but the inducible nuclear pool of the enzyme has a wholly different function in resveratrol-exposed cells [21, 43, 45, 46] in which it forms complexes with pERK1/2, p53 and SUMO-1 to act as a co-activator for p53-responsive genes [47].

In the model of resveratrol-induced apoptosis, physiological concentrations of T_4_ prevent serine phosphorylation of p53 and block stilbene-induced cancer cell apoptosis. The nuclear accumulation of COX-2 is also inhibited [21]. These actions on resveratrol action are rapidly initiated at the binding site for thyroid hormone on αvβ3 [42]. The limited clinical success of resveratrol as a cancer chemotherapeutic agent [48] has been attributed to the limited bioavailability of the stilbene. We have speculated instead that endogenous (host) circulating thyroid hormone is the principal cause of ineffectiveness of resveratrol in the clinical setting [18]. As noted in the next sections, there are a number of anti-apoptotic mechanisms of thyroid hormone beyond p53 activation in tumor cells.

### TNF-α and Fas and thyroid hormone

The extrinsic apoptosis pathway in cancer cells and nonmalignant cells is initiated by interaction of so-called death-related ligands with specific receptors on the cell surface. TNF-α and Fas ligand are such factors. Thyroid hormone has been shown to suppress apoptosis in trophoblast cells by decreasing expression of *TNF-α* and *FAS* and Fas ligand genes [49]. In the same cells, the hormone decreased the activity of caspase-3, another anti-apoptotic action. In mouse hepatocytes, Sukocheva and Carpenter [27] have shown that T_3_ is anti-apoptotic via interference with TNF-α\Fas mechanisms. The hormone prevented specific caspase activation and DNA fragmentation and blocked TNF-induced cell acidification. That acidification is an important component of the thyroid hormone-TNF-α apoptotic pathway was shown in these studies by pharmacologic inhibition of the Na^+^/H^+^ exchanger (NHE1) that eliminated the anti-apoptotic effect of T_3_. The exchanger is activated by T_3_ [50]. The intracellular alkalinization that can be induced by thyroid hormone may also be relevant to the function of P-glycoprotein (P-gp; MDR1), a plasma membrane pump that exports a number of cancer chemotherapeutic agents (see below).

### Thyroid hormone action via αvβ3 on transcription of apoptosis-relevant genes

We know that the plasma membrane thyroid hormone/hormone analogue receptor site on the integrin controls transcription of apoptosis-promoting genes, such as *CASP2* and *BCL2L14* (Bcl-2-like protein 14), and anti-apoptosis genes, such as X-linked inhibitor of apoptosis (*XIAP*) and myeloid cell leukemia 1 (*MCL1*) [29, 34]. Tetrac blocks binding of both T_4_ and T_3_ to integrin αvβ3 [7, 25]. Tetrac and a nanoparticulate tetrac formulation (Nanotetrac) that is excluded from the cell interior and acts exclusively at the integrin cause suppression of *XIAP* and *MCL1* gene expression and induce transcription of *CASP2* and *BCL2L14* and other pro-apoptotic genes [7, 51]. Such pro-apoptotic actions of the T_4_ inhibitor (tetrac/Nanotetrac) are consistent with the demonstrated anti-apoptotic activity of T_4_, and direct examination of the actions of T_4_ or T_3_ on apoptosis-relevant genes has indeed shown this to be the case. For example, T_3_ decreases transcription of pro-apoptotic *BAD* (Bcl-2-associated death promoter) and upregulates anti-apoptotic *BCL2* in nonmalignant cells [52] without affecting pro-apoptotic *BAX* (Bcl-2-associated X protein) or *AIF* (apoptosis inducing factor). Thyroid hormone also decreases cellular abundance of caspases, e.g., caspase-3, and of *BAX* [32, 39, 53], and increased the expression of *XIAP* [54]. As mentioned above, the hormone also increases expression of *HIF1α*, the gene product of which has complex anti-apoptotic functions [35, 55, 56]. The tumor growth arrest that is achieved with imposition clinically of the euthyroid hypothyroxinemic state mentioned above [23] is also consistent with removal of the anti-apoptotic influence of T_4_.

### Apoptosis-relevant action of thyroid hormone on mitochondria

The recognition of the existence of anti-apoptotic activity of the hormone and of known effects of the hormone on mitochondrial activity [1] raised the possibility that mitochondria-directed (intrinsic pathway) apoptosis might be affected by iodothyronines. Mukherjee and co-workers [57] have recently shown that T_3_ administration rescues hypothyroid rat liver cells from apoptosis induced by oxidative stress that causes inner mitochondrial membrane damage. Thus, thyroid hormone is anti-apoptotic in this model system.

Activation of caspases that begins in the extrinsic apoptosis pathway converges with the intrinsic pathway at mitochondria, with disruption of the mitochondrial membrane and freeing of cytochrome c, a hallmark event in apoptosis. Cytochrome c release may in turn further activate caspases. Support by thyroid hormone of expression of the *BCL2* family of genes [58, 59] also has anti-apoptotic consequences. A principal Bcl-2-related protein, MCL1, resides in the outer mitochondrial membrane and binds Bak (Bcl-2 homologous antagonist/killer) and Bax proteins, thus preventing their destabilization of the membrane. Thyroid hormone activates expression of *MCL1* [59], preventing mitochondrial membrane destabilization and the formation of channels by which mitochondrial cytochrome c is released and apoptosis induced. T_4_ downregulates expression of the *BAX* gene [58], as noted above, whose gene product is pro-apoptotic at the mitochondrion.

### Thyroid hormone and residence time in cancer cells of anti-apoptotic chemotherapeutic agents

Acting at the thyroid hormone receptor on integrin αvβ3, the hormone receptor antagonist, tetrac, has been shown to increase residence time in tumor cells of a variety of cancer chemotherapeutic agents [60]. Among these agents are agents with pro-apoptotic properties, such as paclitaxel, etoposide and doxorubicin. These drugs are subject to export from cells by the P-glycoprotein (P-gp; MDR1, ABCB1) membrane pump and expression of the *MDR1* gene and pump function are both known to be increased by thyroid hormone [61, 62]. Thus, an ‘anti-apoptotic’ action of the hormone may involve shortening of intracellular residence time and effectiveness of pro-apoptotic chemotherapeutic agents. We attribute the effect of tetrac to prolong residence time in cancer cells of doxorubicin and paclitaxel—increasing drug efficacy—to blockade of the thyroid hormone effect on MDR1. Hypoxia-induced multidrug resistance may also be supported by thyroid hormone via its action on HIF-1α [63].

That the hormonal effect on intracellular concentrations of pro-apoptotic chemotherapeutic agents is more complicated than regulation of MDR1 pump activity is emphasized by the example of cisplatin. Cisplatin is not a ligand of MDR1, but its intracellular concentration is lower in thyroid hormone-treated cells than in control cells [60]. This effect of the hormone may involve increased activity of the organic cation transporter that imports cisplatin into cancer cells, but this possibility has not been examined experimentally.

### Involvement of TRs in anti-apoptotic action of thyroid hormone

The foregoing review has dealt with anti-apoptotic activity of thyroid hormone that is initiated by nongenomic mechanisms. However, in human hepatoma cells that overexpress TRα, exposure to T_3_ has been shown to induce resistance to apoptosis [38]. This effect may depend at least in part upon increased expression of *Bcl-xL*. Interestingly, these cells were resistant to apoptosis despite increased transcription of tumor necrosis factor (TNF)-related apoptosis-inducing ligand (*TRAIL*) and, in fact, the enhanced production of TRAIL was associated with increased, rather than decreased, metastatic potential of the cells. Another TR-related mechanism by which thyroid hormone is anti-apoptotic involves mitochondria studied in nonmalignant cells by Saelim and co-workers [64]. Here, at 10^−7^ M in *Xenopus* oocytes, T_3_ targeted to mitochondria by a short form of TRβ1 decreased apoptosis dependent upon cytochrome c release. Similar results were obtained in primate kidney (CV-1) cells. Thus, several TR-dependent mechanisms exist by which T_3_ may be anti-apoptotic, but demonstration of the existence of these mechanisms required supraphysiologic concentrations of T_3_.

In contrast, the liganded wild-type TRβ has thyroid cancer suppressor activity, as shown by Cheng and associates [65], and the receptor may induce apoptosis [66]. Multiple mechanisms are involved in tumor suppressor/pro-apoptosis effects, including downregulation of β-catenin expression and rescue by a nongenomic mechanism of p53 from binding to/inactivation by SV40 large T antigen (SV40Tag). The cellular Src kinase (cSrc) phosphorylation site on TRβ that confers tumor suppressor behavior has been identified [67], as have specific miRNAs that contribute to TRβ-directed decreases in cancer cell proliferation and invasiveness [68]. Mutated TRβ loses tumor suppressor activity and may increase cell proliferation and inhibit apoptosis [69]. Others have shown that T_3_ at very high concentrations (10^−6^ M) induces apoptosis in human breast cancer cells [70], apparently via TRβ-dependent downregulation of expression in tumor cells of anti-apoptotic senescence marker protein-30 gene (*SMP30*). Thus, the anti-apoptotic properties of thyroid hormone expressed by thyroid hormone by a variety of nongenomic mechanisms—most of which originate at integrin αvβ3—are well-substantiated, but the contributions of nuclear receptors TRβ and TRα to regulation of apoptosis are varied in result and appear to depend in part upon the model systems used.

## DISCUSSION

Tightly regulated apoptosis is essential to normal tissue development, growth and maintenance of mass. In contrast, the state of anti-apoptosis in cancer cells permits tumor growth, and a number of pro-apoptotic chemotherapeutic agents have been developed to oppose cancer cells’ anti-apoptotic defense mechanisms. Thyroid hormone is an essential factor in the regulation of energy and protein metabolism in nonmalignant cells and normal tissues [1]. In the setting of cancer, however, thyroid hormone, particularly T_4_, acts via its cell surface receptor on integrin αvβ3 as a proliferative factor and is pro-angiogenic [1, 13]. Results of *in vitro* and of human xenograft studies support these roles of the hormone [19, 22, 25, 71]. Clinical support for such observations comes from medical interventions to reduce circulating levels of thyroid hormone [23, 72], from effects of hypothyroidism secondary to tyrosine kinase inhibitor use in patients with renal cell carcinoma [73, 74] and from the effects of spontaneous hypothyroidism on the clinical behavior of breast cancer [75].

That thyroid hormone is desirably anti-apoptotic in nonmalignant cells has been emphasized by other authors [58, 76, 77]. We have previously reported the presence of anti-apoptotic activity of thyroid hormone in cancer cells, a finding that serves to engage the hormone in cancer cell defense [20, 21, 41]. This action of the hormone was initiated at a cell surface receptor for thyroid hormone integrin αvβ3 and was inhibited by tetrac or tetrac formulations. In the present review, we have examined the molecular mechanisms by which the hormone can be anti-apoptotic and by which tetrac or its nanoparticulate formulation can be pro-apoptotic. What is clear is that multiple components of the extrinsic and intrinsic apoptosis pathways are largely modulated from αvβ3 by thyroid hormone—chiefly, T_4_—at physiological concentrations. These components include expression of genes for Fas ligand, caspases, multiple members of the Bcl-2 family and XIAP (Figure 1). Also affected is phosphorylation of p53. As a result of the congruence of these factors, cytochrome c release by mitochondria is decreased. Thus, a panel of proteins is affected with roles in apoptosis that affect the integrity of the mitochondrion. Finally, the hormone may limit chemotherapy-induced apoptosis by shortening intracellular residence time of anticancer drugs that are pro-apoptotic. All of these factors are *possible* clinical contributors to chemotherapy resistance, such as that which has been modeled *in vitro* in myeloma cells exposed to bortezomib and thyroid hormone [78].

How broadly the anti-apoptotic activity of thyroid hormone may affect chemotherapy is not yet known. It is possible to begin to examine the extent of anti-apoptosis with reduction in circulating levels of host cancer patient T_4_ while maintaining normal blood levels of T_3_ [23]. It may also be possible in the future to block the anti-apoptotic and other cancer support actions of T_4_ at integrin αvβ3 with pharmaceutical agents [7, 21], and monitor effectiveness of existing cancer chemotherapeutic drugs with pro-apoptotic properties.
